# Re-locative guided search optimized self-sparse attention enabled deep learning decoder for quantum error correction

**DOI:** 10.1038/s41598-025-87782-2

**Published:** 2025-01-29

**Authors:** Umesh Uttamrao Shinde, Ravikumar Bandaru

**Affiliations:** Department of Mathematics, School of Advanced Sciences, VIT-AP University, Besides AP Secretariate, Amaravati, Andhra Pradesh 522237 India

**Keywords:** Heavy hexagonal code, Quantum circuits, Error correction, Deep learning, Statistical features, Engineering, Mathematics and computing

## Abstract

Heavy hexagonal coding is a type of quantum error-correcting coding in which the edges and vertices of a low-degree graph are assigned auxiliary and physical qubits. While many topological code decoders have been presented, it is still difficult to construct the optimal decoder due to leakage errors and qubit collision. Therefore, this research proposes a Re-locative Guided Search optimized self-sparse attention-enabled convolutional Neural Network with Long Short-Term Memory (RlGS2-DCNTM) for performing effective error correction in quantum codes. The integration of the self-sparse attention mechanism in the proposed model increases the feature learning ability of the model to selectively focus on informative regions of the input codes. In addition, the use of statistical features computes the statistical properties of the input, thus aiding the model to perform complex tasks effectively. For model tuning, this research utilizes the RIGS nature-inspired algorithm that mimics the re-locative, foraging, and hunting strategies, which avoids local optima problems and improves the convergence speed of the RlGS2-DCNTM for Quantum error correction. When compared with other methods, the proposed RlGS2-DCNTM algorithm offers superior efficacy with a Minimum Mean Squared Error (MSE) of 4.26, Root Mean Squared Error of 2.06, Mean Absolute Error of 1.14 and a maximum correlation and $R^2$ of 0.96 and 0.92 respectively, which shows that the proposed model is highly suitable for real-time error decoding tasks.

## Introduction

Worldwide, industries could transform because of quantum technologies. Utilizing the power of quantum mechanics, several challenging issues were rectified in diverse fields including materials science, machine learning cryptography, logistic optimization, finance aerospace industries, and, pharmaceutical^2,14–16^. The cautious design of numerous interconnected components is necessary to create a quantum computing architecture that can execute large-scale computations^9^ Beyond quantum communication, quantum computers are additional crucial information applications. Because quantum states are non-copyable and cannot be directly measured by quantum computers, error-correcting techniques used in classical computers could not be transferred to quantum computers^8^. Qubits are a key component of quantum computers, just like in classical computers^10,11^. Superposition and entanglement are two instances of quantum mechanical features that set qubits apart from classical bits and allow quantum computers to solve complicated problems that classical computers are unable to handle. However, because of the extreme fragility of quantum states and their susceptibility to outside environmental noise, interactions between qubits and their surroundings are unavoidable and can result in qubit errors and decoherence^1,12,13^.

Using quantum-error-correction (QEC) techniques, which encode several defective physical qubits into a logical quantum state, akin to classical error correction, the errors caused by decoherence can be reduced^3^. Although quantum gates have decreased in the past ten years, they still have extremely high error rates. Therefore, scalable quantum error correction codes (QECCs) are essential, which allow errors to be rectified exponentially while the complexity of encoding and decoding codewords becomes sub-exponentially with the number of qubits^17^. The surface code (SC) is one of the most often used QEC schemes because of its scalable 2-D structure, high error threshold, and requirement for just next-neighbor interactions^3^. However, each qubit is attached to four other qubits on the dense lattice of an SC, which may result in many frequency collisions between them. To address this issue heavy hexagonal code is developed, which connects the vertices of physical qubits using a combination of degree-two and three vertices^1^. By using extra qubits or marked qubits in the syndromic measurement, this method minimizes qubit crosstalk while reducing the number of different frequencies needed for execution, which is one of its primary benefits. The heavy-hexagon code is a hybrid surface and Bacon-Shor code mapped onto a (heavy-) hexagonal lattice. This lattice includes all the ancilla qubits required for fault-tolerant error correction^44^. A quantum computer’s hexagonal structure complements this coding scheme nicely.

Although the heavy hexagonal code achieves error correction by using tight interactions between physical qubits, decoding it is extremely difficult^18^. The need for more intricate neural network (NN) models grows with the decoding distance^1^. Hardware-based decoders that are sufficiently fast have been demonstrated in recent work^19,20^, but there is a dearth of research on the hardware implementation of a decoding solution that could surpass them. NN decoders have garnered significant attention due to their state-of-the-art decoding performance and quick and consistent inference time^3,21–23^. In machine learning, deep quantum error correction refers to the use of deep learning methods to improve the detection and correction of faults in quantum computing. A NN is trained to identify patterns linked to faults in the quantum states^24,25^ and gains the ability to forecast quantum state mistakes during training, which allows it to direct a later error correction procedure^4,26^. Furthermore, quantum error correction loss correction thresholds can be computed using machine learning^7,27,28^. While the aforementioned techniques might be used to create ML decoding for the specific scenario of low redundancy, memory restrictions would still be an issue^2^.

The research aims to design an effective deep learning-based decoder for QEC, which tackles the aforementioned challenges of conventional decoders. The deployment of the self-sparse attention mechanism and the RlGS algorithm offers a sophisticated tool for error decoding. The feature learning ability of the proposed RlGS2-DCNTM model is enhanced using the self-sparse attention mechanism. Additionally, the extraction of statistical features improves the reliability and efficiency of the model. The key highlights of the research are described as follows,

- *Re-locative guided search optimization algorithm* The RlGS algorithm mimics the biological characteristics of birds including unique hunting, and searching traits, which effectively optimizes the learning parameters of the proposed model for effective error detection and correction. The utilization of guided search and re-locative-based moment strategies avoids premature convergence issues and energy depletion problems.
- *Re-locative Guided Search optimized self-sparse attention enabled convolutional Neural Network with Long Short-Term Memory (RIGS2-DCNTM)* The combined RlGS2-DCNTM model with the self-sparse attention mechanism offers superior error decoding performance in heavy hexagonal codes. The robustness of the proposed model for handling complex error patterns can improve fault tolerance in quantum computing systems.

The subsequent sections of the manuscript are organized as follows, the literature review of the existing methods is described in Sects. “Literature review”, and “System model for quantum error correction” details the system model. The working flow of the proposed method with a detailed mathematical description is explained in Sect. “Proposed re-locative guided search optimized self-sparse attention enabled deep learning model for quantum error correction” and the research findings, performance as well as comparative results are discussed in Sect. “Results and discussion”. Finally, the research conclusion with future works is mentioned in Sect. “Conclusion”.

## Literature review

This section reviews the conventional QECC techniques developed for quantum computers and also discusses the challenges and advantages of the existing decoders.

Li et al.^1^ designed an error correction decoder using CNN, which determined the decoding threshold value effectively. Furthermore, the ResNet model architecture was optimized by maximizing the hexagonal code values. When compared with other error correction codes, the hexagonal codes offered significant accessibility. However, the complexity of the hexagonal codes increased concerning the distance between the codes, which limited the real-world applicability of the model. Cruz et al.^2^ initiated a QEC method using a noise guessing approach that decodes the error from the original bits, which was appropriate for time-variant statics. However, the model was only applicable to limited qubit circuits.

A feed-forward NN-based QECC was implemented by Overwater et al.^3^, offered higher decoding performance than the existing methods and also minimized the requirements of tight delay. However, to evade a data blockage, the established method necessitated a large margin, which increased time complexity. Shinde and Bandaru^4^ designed a QEC approach based on an optimized self-adaptive deep learning model, which improved the error correction performance in heavy hexagonal codes. However, the established model did not correct dynamic errors, which impacted the resilience of the deep learning model in real-time applications.

Kim et al.^5^ modeled a biased error correction approach for a heavy hexagon structure, in which the measurement circuit was designed based on three different qubits including flag qubit, data qubit, and syndrome qubit. However, the obtained correlation in the decoder section degraded the performance of the model. Ustün et al.^6^ deployed a single-step parity check (SSPC) gate for QEC. The integration SSPC minimized the number of steps for error correction, thus diminishing the time complexity. Further, in the presence of noise, the SSPC technique resulted in a high perfection rate. Despite its superior performance, the designed technique produced false positives, and it did not detect errors in the code bit.

A deep Q network decoder was established by Ding et al.^7^ to solve data scale problems in the QEC approach. In addition, the Deep Q network effectively learned the relation between the logical error rate and inversion error rate, which offered robust error correction accuracy. However, the unstable disturbances outside the ideal situation affected the physical transmission process. A deep learning-based error suppression algorithm was designed by Cao et al.^8^, which exhibited superior performance in small-distance semion codes. The deep learning approach ensured the code’ degree of freedom and also demonstrated more flexible error correction performance than the conventional approaches. However, the fragile nature of the teleportation method, the implemented error suppression algorithm is limited to short distances.

Wang et al.^28^ created a convolutional neural network (CNN) decoder to correct errors in the toric code. In this ResNet is used to reduce the running time. However, the created model did not correct the errors, which affected the utilization of the model in real-time application. Zhao^34^ established the machine learning models for quantum error correction. The receptive field is enlarged to exploit information from distant ancilla qubits which assists in the significant improvement of quantum error correction. However, due to the presence of unreliable data qubits in the quantum computers, implementing quantum error correction is a challenging one when establishing a stable quantum computer system.

### Challenges

- The CNN-based decoding algorithm poses model complexity during training and testing with intricate noise models, which affects the practical application of the decoders in real quantum computing environments^1^.
- The noise-guessing approach has led to increased errors during the encoding and decoding process. Additionally, the designed approach was only effective for systems with restricted qubit interconnectivity and within the confines of fault-tolerant systems^2^.
- The interference of external factors in the decoder remains a significant challenge in the Q networks^7^.
- The self-adaptive deep learning model exhibited a lack of error resilience, which affected the decoding efficiency of the model in real-time applications.

### Problem statement for quantum error correction

The idea behind quantum error-correcting codes is to convert physical qubits into logical qubits to lessen the impact of noise on qubits^12^. Surface codes are the primary focus of research among the many quantum error-correcting codes that have been suggested and examined. The family of quantum error-correcting codes known as surface codes makes use of the neighborhood interactions between physically organized qubits in a planar arrangement to repair errors^9^. However, the surface code approaches often exhibit certain challenges associated with leakage in errors, unwanted communication between nearby qubits, qubit state preparation, and measurement errors^5^. Therefore, to mitigate these challenges heavy hexagonal code is designed, which offers scalable performance in QEC and also reduces the frequency of conflicts.

Despite its advantages, the traditional methods initiated based on heavy hexagonal code produce limitations regarding model complexity, and lack of fault-tolerant performance^2^. As a result, to address the challenges of existing QEC methods, this research proposes a hybrid deep learning model. In general, the quantum state of *N* qubits is specified in Hilbert space $H_N$ as follows,

1 $$\begin{aligned} H_{N} = \underbrace{S^2 \otimes S^2\otimes ......\otimes S^2}_{\text { n times}} \end{aligned}$$

Where $S^2$ indicates the qubits whose pure state is represented by a unit vector in 2 dimensional Hilbert space, $\equiv$ specifies the up to renormalization factor, $\otimes$ indicates tensor product. The quantum state vector $|\phi \rangle \in H_N$, which is represented as the summation of overall bit strings $z = (z_1,z_2, ....z_N)$, of *N* bits with $z_j\in Z_2$, which represents the set of integers with modulo 2.

2 $$\begin{aligned} |\phi \rangle \in \sum _{z}G_{z} |z\rangle \end{aligned}$$

Where *G* denotes the complex number, such that $\sum _{z} |G_{z}|^2 =1$. The research aims to introduce a deep learning model to achieve effective error correction, given an error factor *e* to identify a subset *G* of the state and quantum operation $\phi$. The proposed model is trained with complex error patterns for correcting them with an improved learning rate. The computational complexity is managed through the self-sparse attention technique. The deployment of the RlGS2-DCNTM model adapts more readily to various error models, thus enhancing efficiency and accuracy over other QEC techniques. The loss function of the RlGS2-DCNTM model is analyzed in terms of MSE.

## System model for quantum error correction

**Fig. 1:**
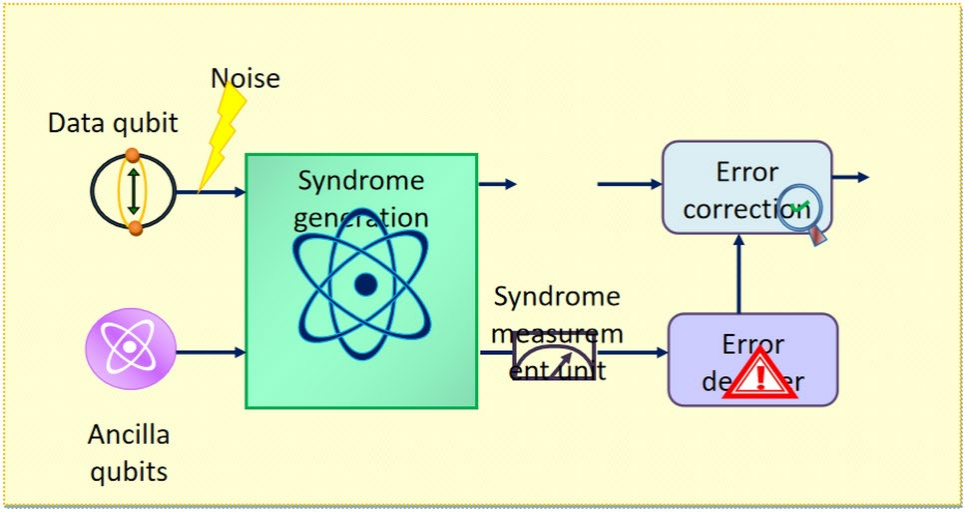
System model for QEC.

QEC remains a significant aspect of quantum computing, which protects quantum information from errors. The system Model for QEC is illustrated in Fig. 1, which comprises several integral components including qubits, syndrome generation, syndrome measurement, error decoder, and error correction. The qubits and ancilla qubits are the basic units of quantum circuits, which measure the quantum state of the circuit. The error is added to the data qubit and the syndrome generation block is responsible for extracting information related to errors. In addition, the syndrome measurement unit is used to determine the presence of errors in the codes by comparing the expected state to the actual state. The error decoder effectively processes the syndrome information to find out the occurrences of errors. Once the type of error is determined, the error correction module applies precise operations to the qubits and corrects the errors effectively.

## Proposed re-locative guided search optimized self-sparse attention enabled deep learning model for quantum error correction

**Fig. 2:**
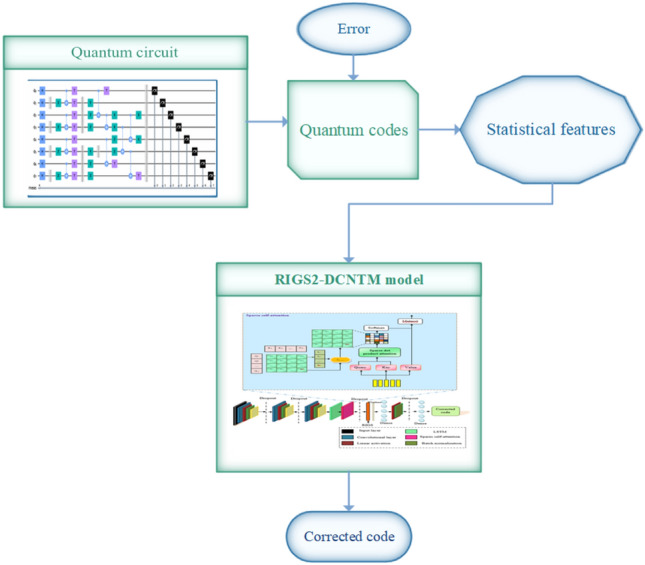
Flow diagram of the RIGS-DCNTM model for QEC.

Deep learning is an effective decoding scheme for topological codes, and in this research, a deep learning-based RlGS2-DCNTM decoder model is proposed for correcting errors in heavy hexagonal codes. Initially, the quantum circuit is simulated using the Qiskit tool, and the code is extracted from the circuits in binary format, in which the errors are added to the code. From the quantum codes, the statistical features are extracted, which enhances the error decoding performance of the model. The extracted features are subjected to the proposed RlGS2-DCNTM model, which integrates the benefits of the self-sparse attention mechanism and the deep learning model. Furthermore, to optimize the hyperparameters of the proposed decoder model the RIGS algorithm is developed, which mimics the biological characteristics of hummingbirds, red kites, and Secretary birds. As a result, the proposed decoder model can effectively correct errors in heavy hexagonal quantum codes, making them more reliable for all quantum computing applications. The schematic illustration of the proposed decoder model is depicted in Fig. 2.

### Simulation and overview of quantum codes

Quantum circuits use a series of quantum gates to carry out computations, in which qubits are the integral parts that represent a pair of logical 1 and 0. In general, QEC is a crucial factor, which prevents quantum information from decoherence and quantum noises. In this research for QEC, the classes and modules of the Qiskit simulator are used to simulate the quantum circuits. Qiskit is an open-source software library that covers the entire stack of quantum computing, from simulation and emulation to application-level algorithms and the actual interaction with the IBM Q hardware. Terra, Aqua, Aer, and Ignis are the four classical elements that are used to identify the four libraries in which the tool itself is organized^29^. Several quantum gates are established in each circuit to carry out particular computational functions. Here, parametrized and customized instruction sets are also examined, increasing the diversity and usefulness of the datasets by enabling a range of circuits to reflect various hardware limits or computing requirements, which evaluates the model’s capacity to work with quantum circuits or build circuits that satisfy particular functional requirements^30^. In this research, the quantum system of the multiple qubit in density matrix form can be denoted as,

3 $$\begin{aligned} \rho = \sum _{i=1}^{N} P_iE_i\sigma E_i^{'} \end{aligned}$$

Where, $P_i$ denotes probability, $E_i$ denotes error patterns. The quantum systems with *N* qubits are referred to as the arbitrary superposition of the $2^N$ basis states. Qubits are represented by wires and controlled by quantum operations (quantum gates) in the circuit model of quantum computation.A quantum circuit simulated using a Qiskit simulator *B* with *f* particular gates is represented by $B = (\zeta _{0},\zeta _{1},\dots ,\zeta _{f-1})$ and they operate on *N* qubits, in which quantum gate acting on a subset is represented by each $\zeta _{i}$ of *N* qubits. Typically, in a quantum circuit, the horizontal line represents the qubit wires, a variety of symbols are used to draw the qubits, and progression is assumed that time passes from left to right.

Among others, heavy hexagonal codes get immense attention, which is the combination of Bacon–Shor codes and Surface codes. The surface code is created by organizing and combining square metrics of four dimensions on a two-dimensional plane. In contrast with surface codes, the heavy hexagonal codes have less connectivity to the neighboring qubits. Put otherwise, it is impossible for any one of the heavy-hexagon structure’s qubits to directly entangle with four qubits. To address the problem, additional auxiliary qubits are employed, which are referred to as flag qubits that allow certain qubits to communicate indirectly with four distinct qubits^31^. Thus, to implement a surface code in a heavy hexagon, three types of qubits are needed: data qubits to construct logical qubit states, syndrome qubits to identify faults, and flag qubits to connect data qubits and the syndrome qubits. The heavy hexagonal codes utilize a set of gauge operators to bring codeword to a subsystem. The stabilizer group of the heavy hexagonal codes is represented using the tensor product of one-dimensional Pauli operators $P_{N}$, which is signified as follows,

4 $$\begin{aligned} P_{N} = \{ \mu _{1} \otimes \mu _{2} \otimes .......\otimes \mu _{N}| \mu _{i} \in \{I,X,Z, Y \}(1\le i \le N)\} \end{aligned}$$

**Fig. 3:**
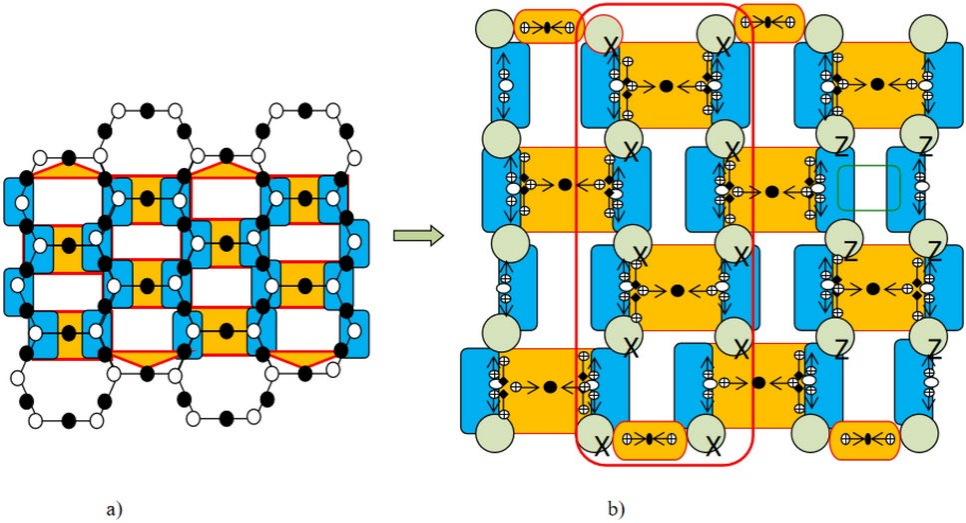
(**a**) The lattice representation of heavy hexagonal code and (**b**) Square lattice structure layout of heavy hexagonal code.

Where *X*, *Y*, *Z* indicates the type of stabilizer codes, and *I* represents the identity gate. The lattice representation of heavy hexagonal code is shown in Fig. 3. The heavy hexagonal codes mainly comprise two regions, in which the region marked with orange color indicates the weight four *X* type and weight two *X* type gauge operators, as well as the blue region comprises weight two *Z* type gauge operators. In addition, the phase error and bit flips are rectified using the *X* type and *Z* type gauge operator. In this research, the codes generated from the quantum circuit are mathematically represented $D = \{d_1,d_2,.....,d_{x},....,d_\eta \}$, in which each binary code is the eight-bit length and $\eta$ denotes the number of codes generated from the circuit.

### Data acquisition for error decoding

In this research, the quantum circuits are modeled using the Qiskit simulator, and the quantum codes are extracted in the binary format $D = \{d_1,d_2,.....,d_{x},....,d_\eta \}$, which is converted into their respective decimal values. The mathematical formulation of the converted decimal values $D_{dcb}$ is mentioned as follows,

5 $$\begin{aligned} D_{dcb} = \{\bar{\omega _{1}},\bar{\omega _{2}},\bar{\omega _{3}},....,\bar{\omega _{x}},...\bar{\omega _{\eta }}\} \end{aligned}$$

The conversion of binary $d_{x}$ to decimal format $\bar{\omega _{x}}$ is an integral step, as it translates the results of quantum computations into an analyzed format. Additionally, the errors (*e*) are added to the codes which are in decimal format and are mentioned using the following equation.

6 $$\begin{aligned} D_{dcb}^{error} = \{\bar{\omega _{e1}},\bar{\omega _{e2}},\bar{\omega _{ee3}},....,\bar{\omega _{x}},...\bar{\omega _{e\eta }}\} \end{aligned}$$

Where $D_{dcb}^{error}$ represents the set of codes embedded with error and the error code $\bar{\omega _{ex}}$ is in the time series format, which is further provided into the statistical feature extraction process with a dimension of $(\eta ,12)$.

### Statistical feature extraction

Statistical features aid as a valuable technique for QEC tasks, in which it analyzes the qualitative characteristics of quantum codes. In this research, the statistical features including variance, mean, median, standard deviation, kurtosis, entropy, skewness, sum, minimum, maximum, sum minimum, and sum maximum are extracted from the error code $\bar{\omega _{ex}}$. The mean evaluates the average value of *n* numbers, and the dispersion or spread of the quantum codes along with its squared difference from the mean is evaluated using variance. The median features evaluate the middle value of the error codes. The difference of each data point from the mean value is evaluated in terms of standard deviation. The higher value of standard deviation measures the spread of data points over a wider region^32^. The tailedness of the data distribution can be quantified through the kurtosis features^33^. Further, the entropy features measure the degree of randomness or unpredictability within the input code. The deviations of data from the normal distribution are analyzed using skewness. In mathematical terms, skewness can be measured as the sum of cubed deviations from the mean, divided by cubed standard deviation and multiplied by the number of applications. The sum represents the mathematical feature, which adds values in the range. The maximum and minimum values of the given error code can be evaluated using the maximum and minimum features. The statistical features are beneficial in evaluating the efficiency and reliability of QEC schemes, ultimately contributing to the advancement of robust quantum error decoding.

**Table 1:** Statistical features.

Sr. No.	Feature	Formulae
1	Mean $(W_{mean})$	$W _{mean} = \frac{\sum _{x=1}^{n} \bar{\omega _{ex}}}{n}$
2	Variance $(W_{var})$	$W_{var} = \sum _{x=1}^{n} (\bar{\omega _{ex}}- W_{mean})^2$
3	Median $(W_{med})$	Middle value
4	Standard deviation $(W_{SD})$	$W_{SD} = \sqrt{\frac{\sum _{x=1}^{n} (\bar{\omega _{ex}}- W_{mean})^2}{n-1}}$
5	Kurtosis $(W_{kur})$	$W_{kur} = \frac{\sum (\bar{\omega _{ex}}- W_{mean})^4}{n(W_{var})^4}$
6	Entropy $(W_{Ent})$	$W_{ent} = -\sum _{x=1}^{n} \rho (x) log_{2}\rho (x)$
7	Skewness $(W_{skew})$	$W_{skew} = \frac{\sum (\bar{\omega _{ex}}- W_{mean})^3}{(n-1)(W_{SD})^3}$
8	Sum $(W_{S})$	The function is used to add numbers in a sequence.
9	Maximum $(W_{Max})$	Maximum value in the given error code
10	Minimum $(W_{min})$	Minimum value in the given error code
11	Sum Maximum $(W_{SM})$	Evaluates the sum of maximum values
12	Sum Minimum $(W_{S min})$	Evaluate the sum of Minimum values.

The mathematical formulation of the statistical features is shown in Table 1, in which $\rho (x)$ indicates the occurrence probability. The features are combined as follows,

7 $$\begin{aligned} W = ||W_{mean}||W_{var}||W_{med}||W_{SD}||W_{kur}||W_{Ent}||W_{Skew}||W_{S}||W_{max}||W_{min}||W_{SM}||W_{S min}|| \end{aligned}$$

The combined statistical features *W* with dimensions of $(\eta ,12)$ are provided in the proposed model for effective error decoding from the input quantum codes.

### Proposed RIGS2-DCNTM model for quantum error correction

Error decoding is a significant aspect of quantum computing, which addresses the problems of maintaining the integrity of quantum information. The research introduces a hybrid deep learning-based error decoding model known as RIGS2-DCNTM, which integrates the advantages of self-sparse attention with CNN and LSTM models. Further, the combination of the deep learning and attention mechanism enhances the error decoding performance, which also addresses complex errors in the heavy hexagonal codes. In recent times, several classical and artificial intelligence-based error decoding techniques have been implemented that provide a foundation for error detection and correctability, which are also applicable in the quantum realm. The classical codes cause measurement limitations, which prohibit the duplication of quantum states^9^. In addition, the machine learning-based QEC techniques create over-smoothing problems and necessitate additional ancilla qubits for error correction^34^. The reinforcement learning-based QEC technique introduced in^35^ served as a minimum weight-matching decoder, however, the generalization performance of the model was limited, thus lacking the performance in advanced noise models.

**Fig. 4:**
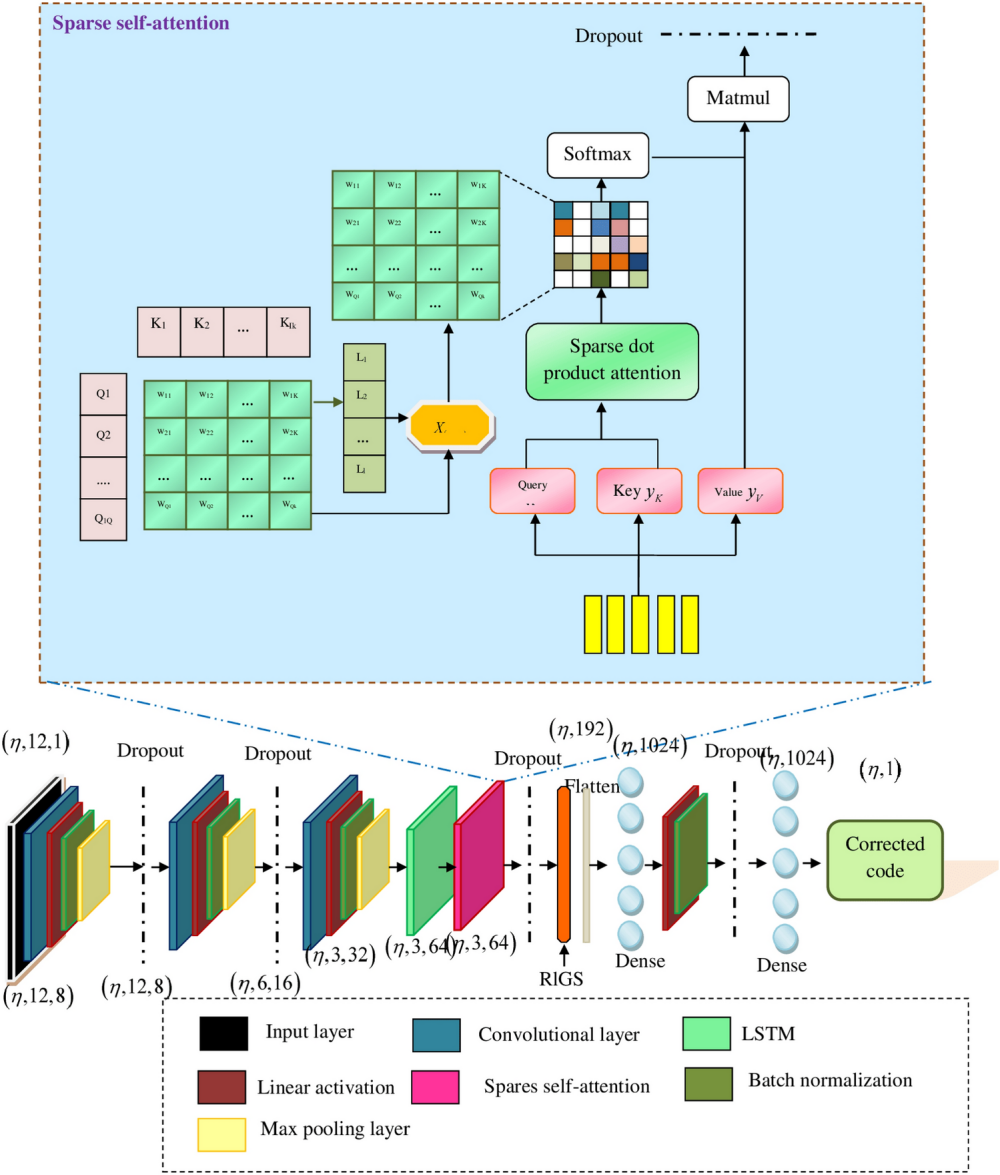
Flow diagram of the RIGS-DCNTM model for QEC.

Therefore, to overcome the aforementioned shortcomings of the conventional error decoding techniques, this research proposes a RlGS2-DCNTM model. The RlGS2-DCNTM model consists of the input layer, three convolutional blocks with a single LSTM, self sparse attention layer, a flattened layer, and dense layers, which is illustrated in Fig. 4. Initially, the concatenated features *W* are subjected to the input layer, which is responsible for the initial representation of encoded codes. The input layer further transfers the features into the convolutional block, in which each convolutional block comprises convolutional, max pooling, linear activation, and a Batch Normalization (BN) layer. The convolutional layers in the RlGS2-DCNTM model are made up of several filters, which convolve the features of input as well as extract the most significant features for further error decoding and correction process^36^. In addition, the convolutional layer transmits and maintains the error information among the qubits. The $\mu ^{th}$ feature map of $\nu ^{th}$ convolutional layer $x_{\mu }^{\nu }$ is mathematically formulated based on the following equation,

8 $$\begin{aligned} x_{\mu }^{\nu } = \omega _{\mu }^{\nu } W + \theta _{\mu }^{\nu } \end{aligned}$$

Where $\omega _{\mu }^{\nu },\theta _{\mu }^{\nu }$ indicates the layer weights and biases respectively, the introduction of weight-sharing concept diminishes model complexity problems. To introduce a range of activations in the RlGS2-DCNTM model, the linear activation function is deployed and the output is formulated as

9 $$\begin{aligned} \psi _{\mu }^{\nu } = \psi (x_{\mu }^{\nu }) \end{aligned}$$

Where $\psi$ represents linear activation, in addition, the standardization of activation functions can be performed using the batch normalization function that provides stable and faster training for quantum error decoding as well as error correction. Followed by BN, the max pooling layer is employed to minimize the spatial dimension of feature maps via downsampling. The max pooling operation $H_{p}$ can be evaluated as follows,

10 $$\begin{aligned} H_{p} = max(\psi _{\mu }^{\nu }) \end{aligned}$$

The features obtained from the max pooling layer are subsequently provided into the LSTM layer, in which the LSTM layer is the advanced version of the recurrent neural network that is mainly designed for addressing vanishing gradient issues. RlGS2-DCNTM model contains three main gates, namely input, forget, and output gates^37^. The gates are the integral components that make decisions regarding memory information whether to conserve or ignore. The ability of the proposed model to learn long-range dependencies offers superior advancements, and the weight values assigned for model training preserve the features for a long period. The mathematical representation of forget $U_{fg}$, input $U_{ig}$ , and output $U_{og}$ gates are formulated in the subsequent equations,

11 $$\begin{aligned} U_{fg}= & \epsilon (\omega _{fg}[\lambda _{t-1}, h_{p}] \theta _{fg}) \end{aligned}$$

12 $$\begin{aligned} U_{ig}= & \epsilon (\omega _{ig}[\lambda _{t-1}, h_{p}] \theta _{ig}) \end{aligned}$$

13 $$\begin{aligned} U_{og}= & \epsilon (\omega _{og}[\lambda _{t-1}, h_{p}] \theta _{og}) \end{aligned}$$

Where $\omega _{fg},\omega _{ig},\omega _{og}$ and $\theta _{fg},\theta _{ig},\theta _{og}$ represents the weights and bias functions of the respective gates, $\lambda _{t-1}$ indicates the hidden state of the previous time step, and $\theta$ represents the sigmoid activation. The forget gate is responsible for making final decisions, about whether the information could be stored or deleted from the cell state. The input gate controls information flow, and the cell state $U_{cell}$ stores the information over time, which can mathematically evaluated as follows,

14 $$\begin{aligned} U_{cell} = tanh(\omega _{cell}[\lambda _{t-1}, h_{p}]\theta _{cell}) \end{aligned}$$

where *tanh* signifies the activation, $\omega _{cell,\theta _{cell}}$ denotes the weights and biases of cell state, in addition, the hidden state of the RlGS2-DCNTM model is determined as follows,

15 $$\begin{aligned} \lambda _{t} = U_{og} tanh(U_{cell}) \end{aligned}$$

The output obtained from the LSTM layer *y* is provided into the sparse self-attention layer, which enhances the efficiency as well as global learning ability of the RlGS2-DCNTM model^38^. Initially, the output features of the LSTM layer are separated into several vector values including, key $y_{k}$, query $y_{q}$, and value $y_{v}$. The similarity between the key and query is computed using the scaling dot product formula, which obtains the high dimensional matrix, the attention score $\psi$ for the matrix is computed as,

16 $$\begin{aligned} Y = \psi (y_{Q},y_{k}) = \frac{y_{Q}y_{k}^{T}}{\sqrt{c}} \end{aligned}$$

where *Y* represents the weight matrix, $\sqrt{c}$ denotes the dimension of the matrix, the key principle of the sparse attention technique is to eliminate the low-weight features, thus improving the decoding performance of the RlGS2-DCNTM model. The masking operation $X_{(\nu _{1},\nu _{2})}$ of the attention mechanism selects the significant feature, and the masking operation can be defined as follows,

17 $$\begin{aligned} X_{(\nu _{1},\nu _{2})} = {\left\{ \begin{array}{ll} Y_{(\nu _{1},\nu _{2})} & \text {if } Y_{(\nu _{1},\nu _{2})} \ge L_{\nu _1} \\ -\infty & \text {if } Y_{(\nu _{1},\nu _{2})} \le L_{\nu _{1}} \end{array}\right. } \end{aligned}$$

where $(\nu _{1},\nu _{2})$ specifies the feature position in the matrix *Y*, the standardized score $X^{\%}$ is calculated using the softmax activation, which is formulated as,

18 $$\begin{aligned} X^{\%} = softmax(X_(\nu _{1}, \nu _{2})) \end{aligned}$$

The expected value of sparse distribution can be represented as $\theta$ and it is the product of the standardized score and the value vector, which is described in the subsequent equation

19 $$\begin{aligned} \theta = X^{\%} y_{k} \end{aligned}$$

Finally, the matrix multiplication is performed to obtain the final features, the selected feature weights aid in detecting the errors in quantum codes as well as offer superior decoding performance. The extracted features dimensions are flattened into a single dimension and the dense layer is responsible for predicting error values from the code through linear activation function. The weight parameters of the proposed model are optimized using the RlGS algorithm. The final dense layer with the dimension of $(\eta ,1)$ produces the corrected quantum code.

### Re-locative guided search optimization algorithm for parameter tuning

**Fig. 5:**
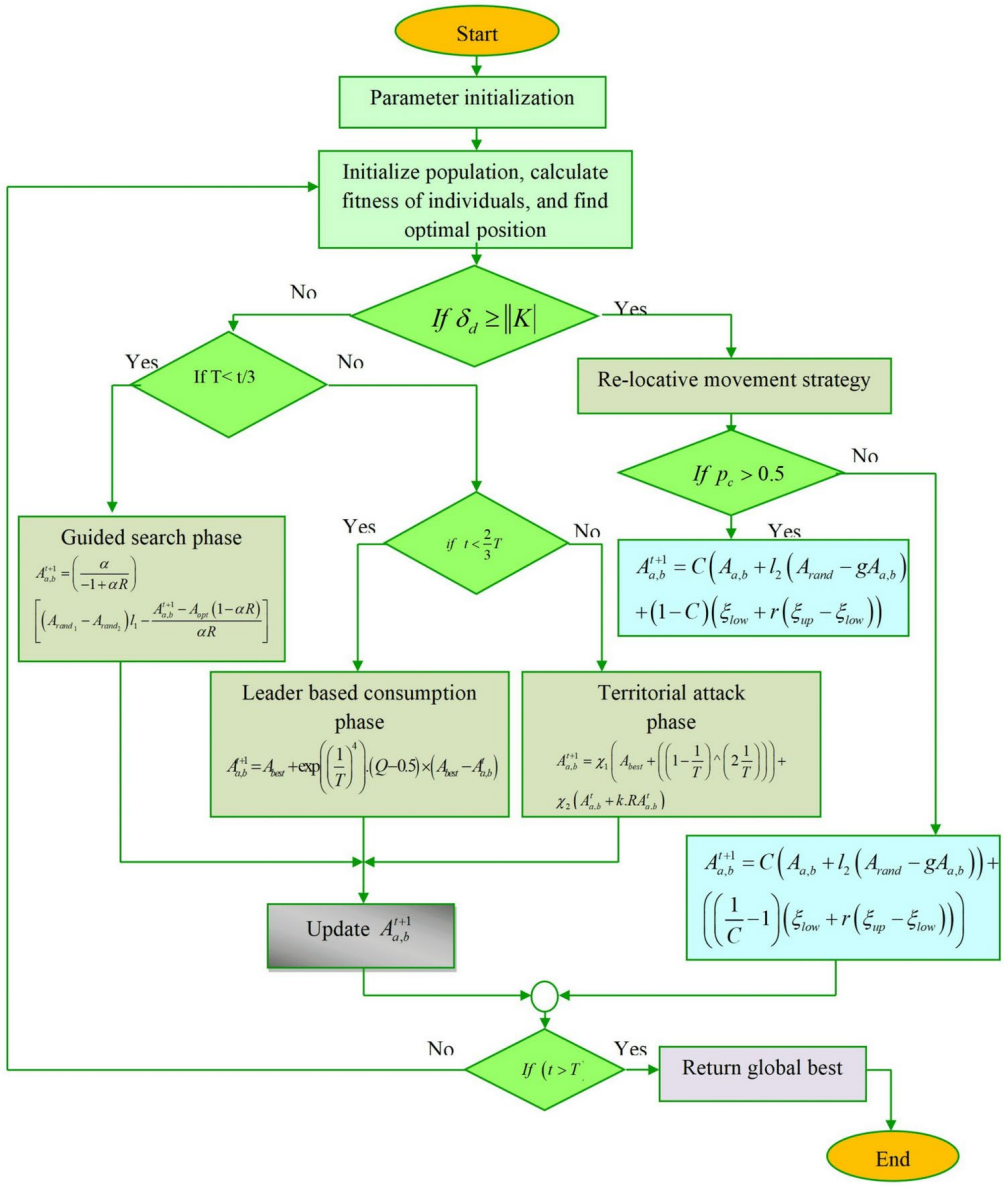
Flow diagram of the RlGS algorithm.

- *Motivation* The proposed RlGS algorithm gets inspiration from the guided search characteristics of hummingbirds with the unique hunting and relocating behaviors of secretary birds and red kits. The RlGS algorithm is mainly designed to optimize the learning parameters of the model and improve the learning rate for obtaining optimal weights. In the context of quantum error decoding, the proposed algorithm effectively detects the errors in the code and avoids local optimal problems.
- *Inspiration* The searching traits mimicking the hummingbirds^39^ serve as significant factors for the RlGS algorithm. In general, the birds perform two types of foraging processes namely self-searching and guided searching. During self-search, the solution searches according to their intellectual behaviors without any intervention from other neighboring solutions. The intellectual behavior majorly depends on the previous search experiences. On the other hand, during the guided search process, the birds follow the guidance information of the leader to obtain optimal targets (error codes). In addition, the unique hunting behaviors of Secretary Birds^40^ improve the convergence speed of the RlGS algorithm without stuck in local optima, which usually walks or trots across grasslands, assuming the stance of a secretary at work by lowering its head and carefully searching the ground for prey concealed in the grass. The amazing thing about Secretary Birds is that they can fight snakes, which makes them a very dangerous foe of reptiles. Additionally, Secretary Bird’s intelligence is demonstrated by its two different techniques of predator avoidance including camouflage and flee. If camouflage is appropriate, to avoid danger, it blends with its surroundings else it will choose to fly or walk quickly to get away from the predator as quickly as possible. Each bird’s position, evaluation function value, point displacement amount, the sound of danger, the sound of unity, a new position, and evaluation function can all be used to model the behavior of a red kite in search of food^41^. The algorithm must first successfully traverse the problem search space to avoid becoming stuck in local optima to get good results. The best solution from the previous iterations is then exploited as it progressively transitions from the exploration to the exploitation phase. The RlGS algorithm applied in the twentieth layer of the proposed model for weight tuning thus enhances the error decoding performance of the model.
- *Solution initialization* The position of each solution in the search space determines the value of decision variables. In the initial implementation of the RlGS algorithm, the solutions are considered as the tuning parameters of the RlGS2-DCNTM model, which is randomly initialized using the following equation,
  20 $$\begin{aligned} A_{a,b} = \zeta _{low} + r(\zeta _{up} - \zeta _{low}) \end{aligned}$$
  where $\zeta _{low},\zeta _{up}$ indicates the lower and upper bounds, *r* represents the random value between 0 and 1, $A_{a,b}$ denotes $a^{th}$ solution in the $b^{th}$ decision variable, in which *a* ranges from 1 to *n*, and *b* ranges from 1 to *m*, *n* denotes the total number of solutions, *m* indicates the decision variable. Furthermore, the population of a candidate solution is randomly generated within the boundary, which is described as follows
  21 $$A_{{pop}} = \left[ {\begin{array}{*{20}l} {A_{{1,1}} } \hfill & {\quad A_{{1,2}} } \hfill & {\quad L} \hfill & {\quad A_{{1,b}} } \hfill & {\quad L} \hfill & {\quad A_{{1,m}} } \hfill \\ {A_{{a,1}} } \hfill & {\quad A_{{2,2}} } \hfill & {\quad L} \hfill & {\quad A_{{2,b}} } \hfill & {\quad L} \hfill & {\quad A_{{2,m}} } \hfill \\ M \hfill & {\quad M} \hfill & {\quad O} \hfill & {\quad M} \hfill & {\quad O} \hfill & {\quad M} \hfill \\ {A_{{a,1}} } \hfill & {\quad A_{{a,2}} } \hfill & {\quad L} \hfill & {\quad A_{{a,b}} } \hfill & {\quad L} \hfill & {\quad A_{{a,m}} } \hfill \\ {A_{{1,1}} } \hfill & {\quad A_{{1,2}} } \hfill & {\quad L} \hfill & {\quad A_{{1,b}} } \hfill & {\quad L} \hfill & {\quad A_{{1,m}} } \hfill \\ \end{array} } \right]_{{n \times m}}$$
- *Fitness evaluation* The objective function of the RlGS algorithm is evaluated based on the values proposed by each solution for the problem variables. The solutions with minimum Mean Squared Error (MSE) can be considered as the fittest solution, which is represented as follows,
  22 $$\begin{aligned} Fit(A_{a,b}^{t}) = Min(MSE(A_{a,b}^{t})) \end{aligned}$$
  The quality of the corresponding solution can be compared with the obtained objective functions for determining the effective solution for the QEC problem. Furthermore, based on danger and unitary factors, the solutions can be updated using different strategies namely re-locative movement strategy, forage and hunt strategy, which are mathematically described in the subsequent section.
- *Case-1: re-locative movement strategy (if* $\delta _{d} \ge ||K||$) The re-locative movement strategy implies that the danger factor $\delta _{d}$ of the solution exceeds the absolute value of the unitary factor ||*K*||, therefore the solution moves to escape from the region and ensures safety from the danger factor. To achieve this, the solutions perform two types of movements known as concealment by environment and run or fleeing. The solution detects the proximity and searches for a suitable concealment environment for effective detection of quantum errors. If not possible, then the solution engages in a rapid fleeing strategy to escape, which is crucial for maintaining the integrity of solutions, particularly when dealing with quantum errors. However, the escaping traits of the algorithm result in entrapment in local regions, since the flight strategy is considered an energy-losing movement than the other one. Therefore, energy consideration for migration from one place to another for error detection is significant. This shows the solution based on the energy factor migrates to the near or far region enough to escape from danger and obtains optimal codes that have errors. The mathematical formulation of the re-locative movement-based solution updation is performed using the following condition.
- *If* $p_{c} > 0.5$ If the conditional probability $p_{c}$ is greater than 0.5, then the solution hides within the safer regions of the problem space
  23 $$\begin{aligned} A_{a,b}^{t+1} = C(A_{a,b} +l_2(A_{rand} - gA_{a,b}) + (1-C)(\zeta _{low} + r(\zeta _{up}-\zeta _{low})) \end{aligned}$$
  where *C* denotes hybridization factor in the range of 0 to 1, $l_2$ indicates the random generation of the array from the normal distribution, *g* denotes random selection integer 1 or 2, and $A_{rand}$ signifies the random candidate solution. The above-mentioned equation is simplified as follows,
  24 $$\begin{aligned} A_{a,b}^{t+1} = C(A_{a,b} +l_2(A_{rand} - gA_{a,b}) + \left( \frac{1}{C} -1\right) (\zeta _{low} + r(\zeta _{up}-\zeta _{low})) \end{aligned}$$
- *Else* If the conditional probability $p_{e}$ is less than 0.5 then the solution performs rapid flee based on the randomly generated array and dynamic perturbation factor, which improves the learning performance of the model for the QEC task. The mathematical representation can be expressed in the following equation
  25 $$\begin{aligned} A_{a,b}^{t+1} = A_{best} + (2Q-1)\left( 1-\frac{1}{T}\right) A_{a,b} \end{aligned}$$
  where $(1-\frac{1}{T})$ signifies the dynamic perturbation factor, *T* represents maximum iteration, and *Q* indicates the Brownian motion that generates an array of dimensions (1, *m*) from a standard normal distribution, $A_{best}$ indicates the best solution. The solution updation can be determined using the following conditions, if the fitness of the current solution is greater than the updated solution then the new updation solution is considered as the best one, or else the current solution remains the best.
  26 $$\begin{aligned} A_{a,b} = {\left\{ \begin{array}{ll} A_{a,b} & \text {if} Fit(A_{a,b}^{t+1} < Fit(A_{a,b} \\ A_{a,b} & \text {Else} \end{array}\right. } \end{aligned}$$
  Collectively, the Re-locative movement strategy is used to find the error factor outside the local value by searching all over the quantum code trained by the model to detect the error accurately.
- *Case (ii): Forage and Hunt strategy ( if* $\delta _{d}< ||K||$*)* When the danger factor is less than the unitary absolute value of the unitary factor, then the solution forage and finds the optimal target solution, and the entire process is divided into guided search space, leader-based consumption phase, and territorial attack phase in three equal time intervals.
- *Subcase -1: Guided Search Phase* ($t< \frac{T}{3}$) n this guided search phase, the solution searches for the targets, and selects the region which has a high chance of optimal targets. The solution makes movements with respect to avoid getting trapped in a local optimum. The individual solutions explore diverse regions of the solution space, which improves the tendency to attain global optimum targets. In the context of error detection in quantum codes, the guided search space prevents premature convergence problems and finds correct error codes. The movement of solutions can be controlled using three different flight factors such as diagonal, axial, and omnidirectional flights. Furthermore, the use of a directional switch factor controls the algorithm to avoid problematic spaces. The flight pattern of the solution *R* should be considered a major factor, the axial flight can be described as follows,
  27 $$\begin{aligned} R^{a}= {\left\{ \begin{array}{ll} 1 & \text {if} a = rand([1,D]) \\ 0 & \text {Else} \end{array}\right. } \end{aligned}$$
  The diagonal flight can be represented using the following equation,
  28 $$\begin{aligned} R^{a}= {\left\{ \begin{array}{ll} 1 & \text {if} a = M(b), b \in [1,q] \\ 0 & \text {Else} \end{array}\right. } \end{aligned}$$
  The omnidirectional flight is indicated as,
  29 $$\begin{aligned} R^{a} = 1,\; i = 1,2,.....D \end{aligned}$$
  In which, *D* represents the dimension space, $M = rand per(q)$ denotes the random permutation of integers from 1 *q* to, the integer *q* can be evaluated as follows,
  30 $$\begin{aligned} q \in [2, \lceil r_1(D-2)\rceil + 1 ] \end{aligned}$$
  where $r_1$ signifies the random number in the range of 0 to 1, and $l_1$ generates random integers within the range of 1 to *D* . The positional update based on the guided search behavior is expressed as follows,
  31 $$\begin{aligned} A_{a,b}^{t+1} = a_{a,b}^{t} + (A_{rand_{1}} - A_{rand_{2}}) l_{1} \end{aligned}$$
  The guided forage based on the optimal target $A_{opt}$ and a guided factor $\alpha$ with respect to the dimension space is formulated as follows,
  32 $$\begin{aligned} A_{a,b}^{t} = \frac{1}{\alpha }A_{a,b}^{t+1} - A_{opt}(1-\alpha R) \end{aligned}$$
  Now substitute Eq. 32 in 31, then the positional updation of the solution can be expressed as,
  33 $$\begin{aligned} A_{a,b}^{t+1} = \frac{A_{a,b}^{t+1} - A_{opt}(1-\alpha R)}{\alpha } + (A_{rand_{1}} - A_{rand_{2}}) l_{1} \end{aligned}$$
  By performing mathematical simplification, the above-mentioned equation is modified as,
  34 $$\begin{aligned} A_{a,b}^{t+1} = \left( \frac{\alpha }{-1+\alpha R}\right) \left[ (A_{raand_{1}} - A_{rand_{2}}) l_{1} - \frac{A_{a,b}^{t+1} - A_{opt} (1-\alpha R)}{\alpha R}\right] \end{aligned}$$
  The updation in the initial time intervals makes the solution to search effective error parts of quantum codes, thus improving the detection performance of the model.
- *Subcase-2: Leader-based consumption Phase* ($\frac{T}{3}< t < \frac{2T}{3}$) : The leader-based consumption phase expresses that the solutions perform movements based on the leader, where the leading solution is selected according to the fitness function, which is described in the following equation,
  35 $$\begin{aligned} A_{best} = A_{a,b} \;\;\;\text {if} Fit(A_{a,b}^{t})< Fit(A_{best}) \end{aligned}$$
  where $A_{best}$ represents the best position of the solution in the current iteration, $Fit(A_{a,b}^{t})$ indicates the fitness of the current solution, based on the leading solution, the other solutions moves towards the target. In addition, the decision-making process also depends on the leader’s solution while making movement nearer to the target solution. The leader-based consumption phase speeds up the algorithm’s convergence to the optimal locations in the search space and assists in avoiding premature convergence to the local optimal. The detection of errors in the codes is increased by taking account of both global and their previous positions. Better outcomes when solving complicated problems are achieved when the unpredictability of Brownian motion *Q* is introduced because it allows individual solutions to more efficiently explore the solution space and offers chances to avoid becoming stuck in local optima. The position updation is formulation using the subsequent equation
  36 $$\begin{aligned} A_{a,b}^{t+1} = A_{best} + exp^{(\frac{1}{T})^4 }(Q-0.5) (A_{best} -A_{a,b}^{t}) \end{aligned}$$
  where *exp* denotes exponential factor, the best position of the optimal target can be determined according to the following conditions,
  37 $$\begin{aligned} A_{a,b} = {\left\{ \begin{array}{ll} A_{a,b}^{t+1} & \text {if} \; Fit(A_{a,b}^{t+1}) < Fit(A_{a,b}) \\ A_{a,b} & else \end{array}\right. } \end{aligned}$$
- *Subcase-3: Territorial attack phase* ($t > \frac{2T}{3}$) During this phase, the solution makes use of its experience in finding optimal solutions to wait for the solution to perform error detection on which opportune movement. But along with that the solution always makes attention to any other targets with already found target. In the context of QEC, the solution that chooses to move to another place of the existing position may have some obstacle factors. The situation of searching with its region for the optimal target is considered an adaptive movement strategy of the solution. The mathematical representation of positional update is denoted as,
  38 $$\begin{aligned} A_{a,b}^{t+1} = \chi _{1} (A_{best} + \left( \left( 1-\frac{1}{T}\right) ^{\frac{2}{T}}\right) + \chi _{2} (A_{a,b}^{t} + kRA_{a,b}^{t}) \end{aligned}$$
  where $\chi _{1}$ and $\chi _{2}$ represents the flexible factors $\chi _{1} + \chi _{2} = 1$, *k* denotes the territorial factor, which is the normal distribution *n*(0, 1) with a standard deviation of 1 and a mean of 0.
- *Termination*When the algorithm meets the termination criteria $t> t_{max}$, the RlGS algorithm stops its iteration and declares the global best solution and end. The overall optimization of re-locative movement and foraging as well as hunting strategies modeled for error detection throughout the quantum codes and finding the optimal error in the current space making equilibrium between the global and local searching strategies. The detailed flow diagram of the proposed algorithm is illustrated in Fig. 5.

## Results and discussion

The subsequent section explains the experimental outcomes and decoding performance of the RlGS2-DCNTM model analyzed using several performance metrics with two different quantum circuits. In addition, the experimental setup details and the simulated circuit details are discussed in the subsequent section.

### Experimental setup

The research execution can be performed using PyCharm software in a Windows 11 operating system with 16 GB RAM and 120 GB ROM. For error decoding, initially, the quantum circuit is generated using the Qiskit simulator. From the circuits, the codes are extracted in binary format which are converted into decimal numbers, and the error value within the range of 0.1 to 0.6 is added to the code. The error code is in time series format, and the statistical features are extracted from the codes and the extracted features are used to train the proposed model.

### Performance metrics

In this research, the QEC performance of the RlGS2-DCNTM model is assessed using performance metrics such as Correlation, MSE, R squared ($R^2$), RMSE, and Mean Absolute Error (MAE). These metrics are significant for determining the efficiency and reliability of QEC codes, which are crucial for the development of fault-tolerant computers.

### Experimental analysis

**Fig. 6:**
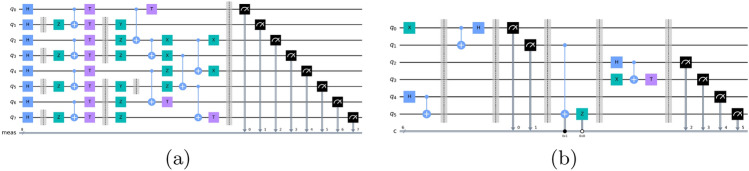
(**a**) Simulated Quantum circuit 1, (**b**) Simulated Quantum circuit 2.

The simulated quantum circuits for QEC using the Qiskit simulator are shown in Fig. 6a and b. In which the first quantum circuit comprises seven qubits with Hadamard gates (H), Pauli X, Y, and Z operators, T gate, CNOT gates, and Measurement units. Similarly, the second quantum circuit comprises five qubits with H, T, CNOT gates and Pauli X, Z operators as well as measurement units.

### Performance analysis

The RlGS2-DCNTM model’s performance is evaluated at various training percentages such as 40, 50, 60, 70, 80, and 90 with varying epochs ranging from 100 to 500. The results determine the learning ability of the model with improved generalization capabilities over time.

#### Performance analysis for circuit-1

**Fig. 7:**
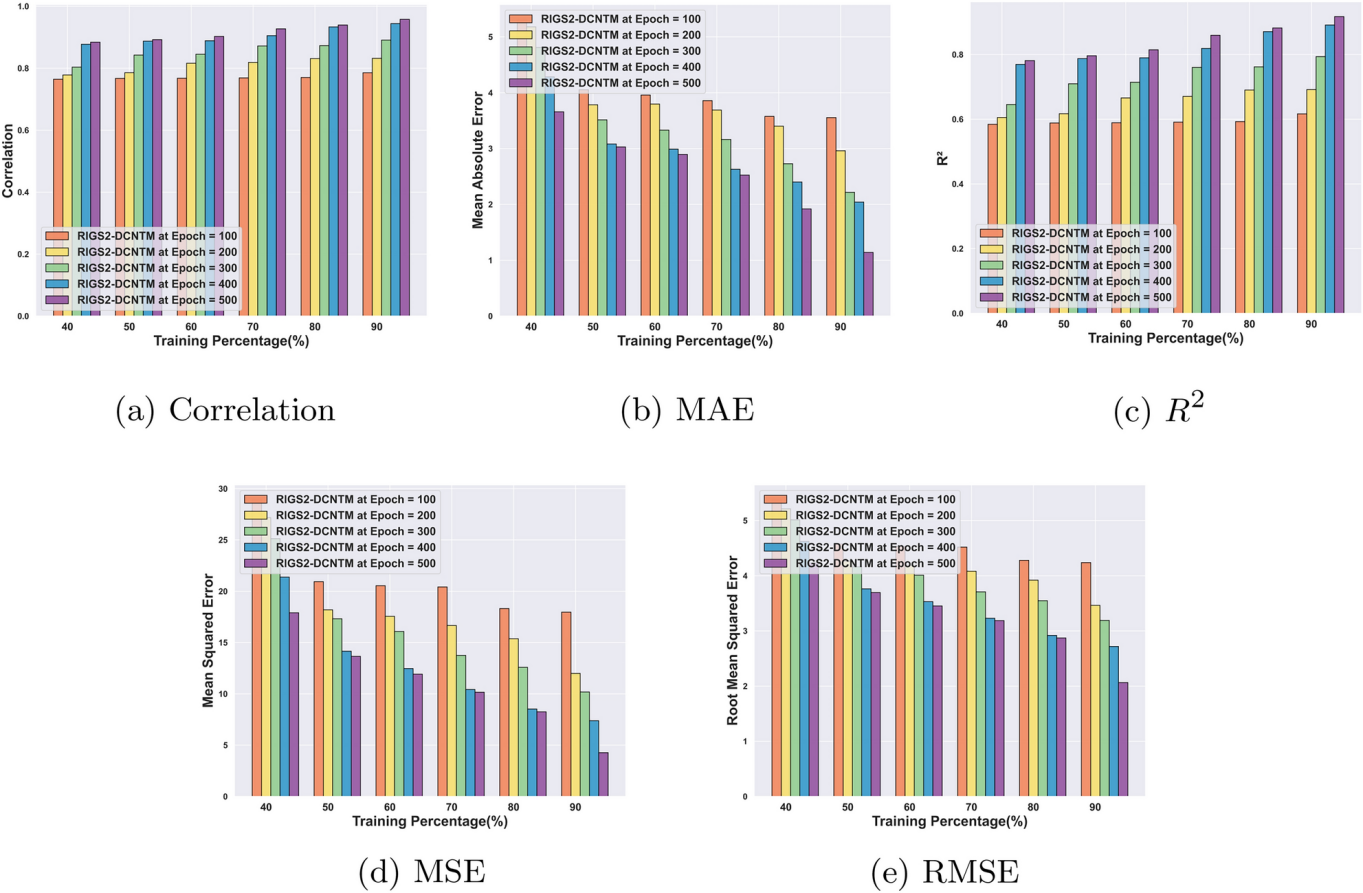
Performance analysis for Circuit 1: (**a**) Correlation, (**b**) MAE, (**c**) $R^2$, (**d**) MSE, (**e**) RMSE.

Figure 7 delineates the performance evaluation outcomes of the RlGS2-DCNTM for circuit 1. For 90% of training with epoch 100, the RlGS2-DCNTM gets a correlation of 0.78, for increasing the number of epochs the RlGS2-DCNTM gets a maximum of 0.95, which is mentioned in Fig. 7a. Thus, the results indicate that the proposed model effectively produces quantum codes with maximum correlation of original codes. Furthermore, Fig. 7b shows that at 90% training, the RlGS2-DCNTM model attains minimum MAE of 3.33, 2.96, 2.21, 2.04, and 1.14 for epoch 100, 200, 300, 400, and 500 respectively. Similarly, in terms of MSE and RMSE, the RlGS2-DCNTM model achieves minimum error values of 4.26 and 2.06 for epoch 500 and TP 90 respectively as depicted in Fig. 7 d and e. The minimum error values of the RlGS2-DCNTM model show superior error decoding performance. As shown in Fig. 7c with 90% of training, the RlGS2-DCNTM decoder attains maximum $R^2$ values of 0.61, 0.69, 0.79, 0.89, and 0.91 for the corresponding epoch values. The superior performance outcomes exhibit that the RlGS2-DCNTM model produces error-free codes, thus enabling the security of the stored information in the qubits. The proposed RIGS algorithm and the self-sparse attention mechanism retain the significant features from the input and improve the convergence speed of the model.

#### Performance analysis for circuit 2

**Fig. 8:**
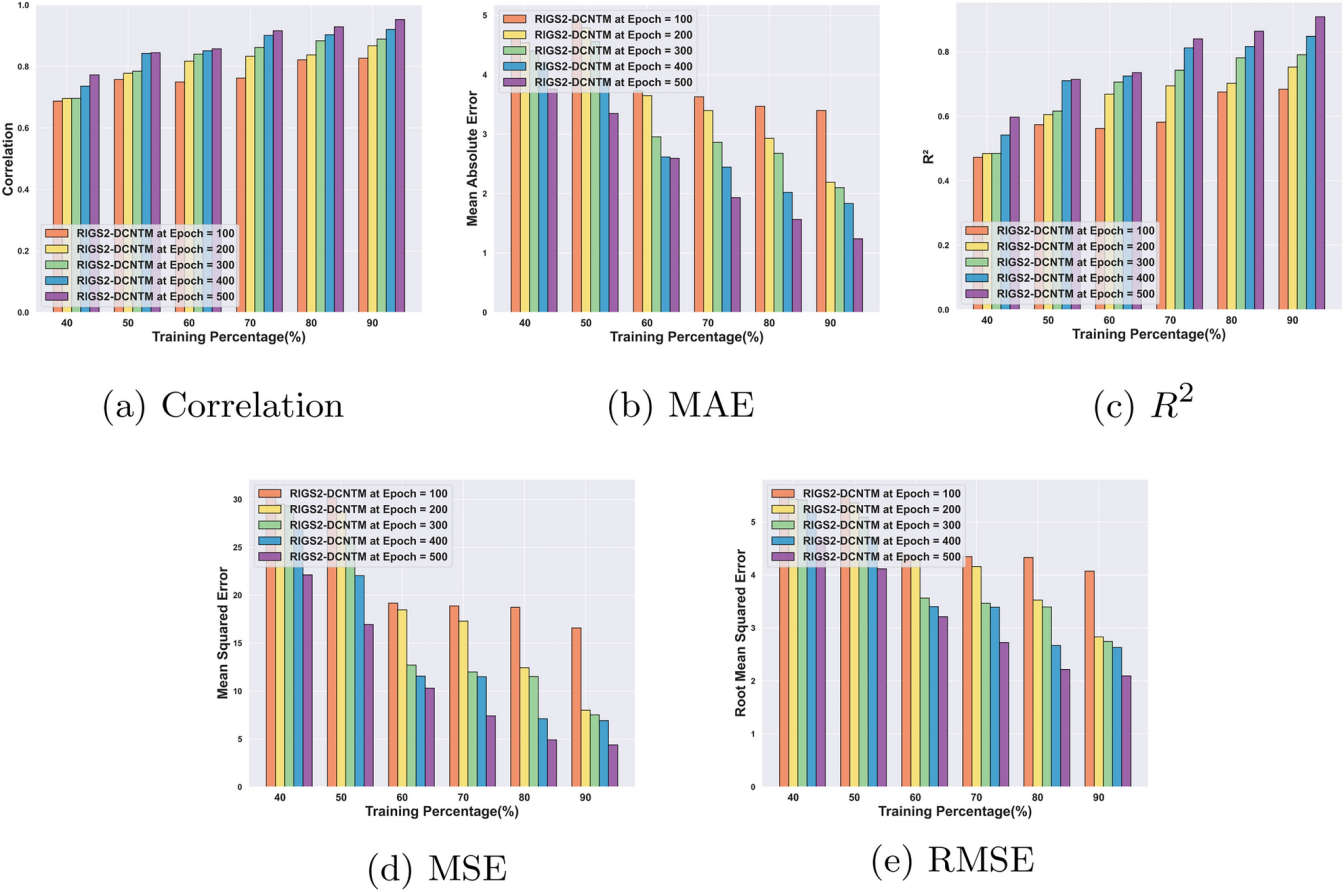
Performance analysis for Circuit 2: (**a**) Correlation, (**b**) MAE, (**c**) $R^2$, (**d**) MSE, (**e**) RMSE.

The evaluation outcomes of the RlGS2-DCNTM model for QEC using circuit 2 are illustrated in Fig. 8. The RlGS2-DCNTM achieves a correlation of 0.82 for 90% of training with epoch 100, and a maximum of 0.95 for 500 epochs, which is represented in Fig. 8a. Accordingly, the results show that the suggested model successfully generates quantum codes with the highest possible correlation of the original codes. Moreover, for epochs 100, 200, 300, 400, and 500, the RlGS2-DCNTM model achieves minimum MAEs of 3.39, 2.18, 2.09, 1.83, and 1.23 at 90% training, shown in Fig. 8b. As per Fig. 8 d, and e the RlGS2-DCNTM model also attains minimum error values of 4.38 and 2.09 for epoch 500 and TP 90, respectively, in terms of MSE and RMSE. The RlGS2-DCNTM model’s higher error decoding capability is demonstrated by its minimal error values. With 90% of training, the RlGS2-DCNTM model attains maximum $R^2$ values of 0.68, 0.75, 0.79, 0.84, and 0.90 for the corresponding epochs, which is illustrated in Fig. 8c.

### Comparative methods

The error decoding performance of the RlGS2-DCNTM model is compared with the traditional error correction techniques such as NN-decoders^3^, QTECNN^8^, ANN^42^, DCNN^1^, Humming Bird Optimization enabled DCNN (HBO-DCNN)^39^, Sparrow Search Algorithm based DCNN^43^, Humming sparrow Optimization based self-adaptive DCNN (HSO-SADCNN)^4^, Red kite optimization enabled DCNTM (RAO-DCNTM)^41^, Humming Bird optimization algorithm based DCNTM (HOA-DCNTM)^39^, and Secretary bird optimization algorithm enabled DCNTM (SBOA-DCNTM)^40^.

#### Comparative analysis for circuit 1

**Fig. 9:**
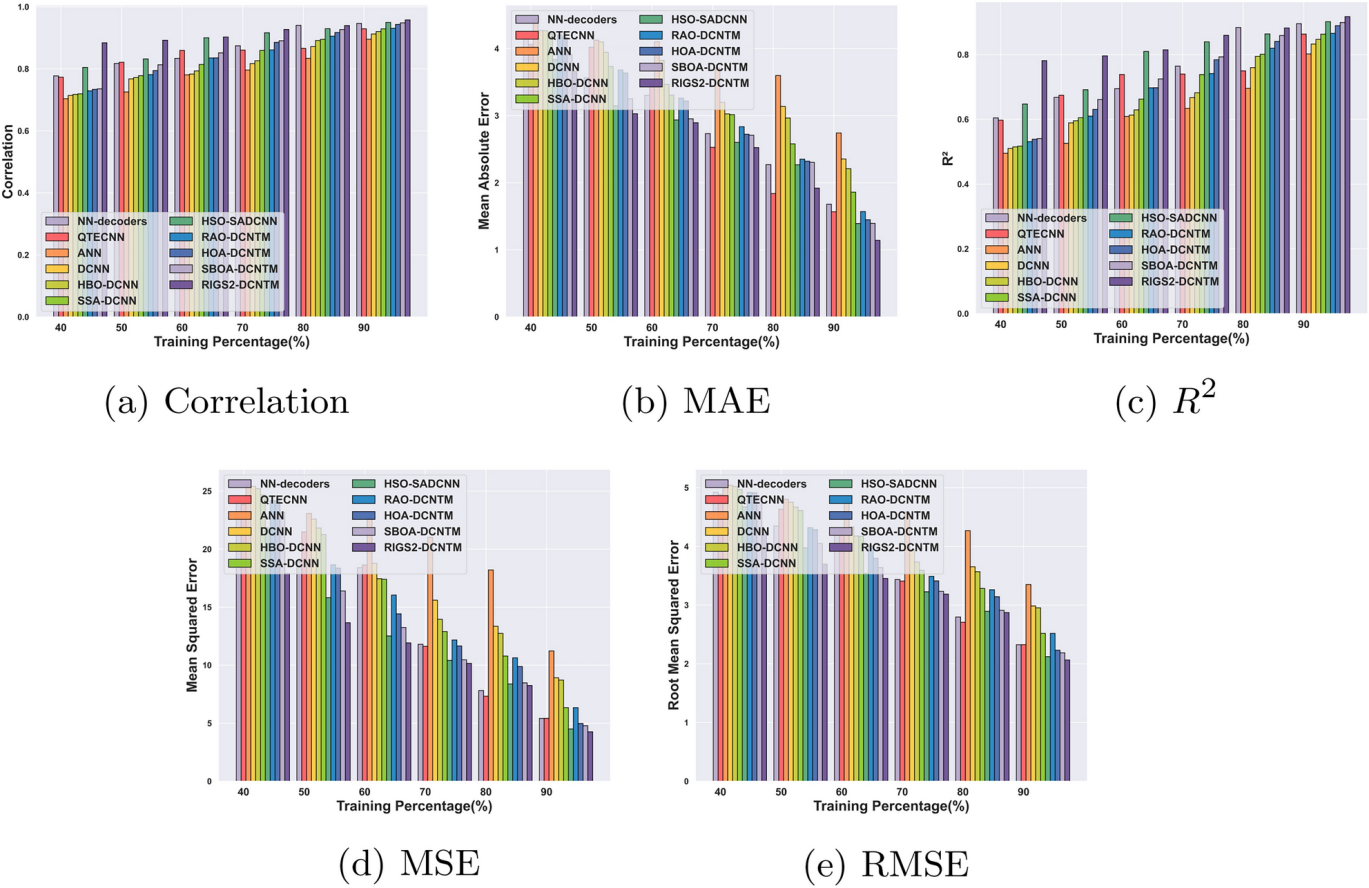
Comparative analysis for Circuit 1: (**a**) Correlation, (**b**) MAE, (**c**) $R^2$, (**d**) MSE, (**e**) RMSE.

The comparative results of the RlGS2-DCNTM model for Circuit 1 are illustrated in Fig. 9. For 90% of training, the RlGS2-DCNTM model attains a maximum correlation of 0.95, which surpasses the existing techniques such as QTECNN, ANN, and DCNN with quite superior differences of 0.028, 0.062, and 0.045 respectively that is depicted in Fig. 9a. With the significant performance difference of 0.54, 0.43, 0.31, 0.25, and 1.21 over the conventional techniques such as NN-decoders, QTECNN, HOA-DCNTM, SBOA-DCNTM, and DCNN, the RlGS2-DCNTM model exhibits minimum MAE of 1.14. For 90% of training that is shown in Fig. 9b. The RlGS2-DCNTM model obtains less error value in terms of MSE of 4.26, which outperforms the conventional SSA-DCNN by 2.08, HSO-SADCNN by 0.24 and ANN by 6.96, which is demonstrated in Fig. 9d. Additionally, Fig. 9c depicts the $R^2$ value of the RlGS2-DCNTM model is 0.92, which shows superior performance deviation of 0.02, 0.05, 0.11, 0.08, and 0.07 over the traditional methods such as NN-decoders, QTECNN, ANN, DCNN and HBO-DCNN respectively. As shown in Fig. 9e, in terms of RMSE, the RlGS2-DCNTM model gets less error value of 2.06, which shows a significant performance deviation of 0.26 over NN-decoders, and 0.88 over HBO-DCNN.

#### Comparative analysis for circuit 2

**Fig. 10:**
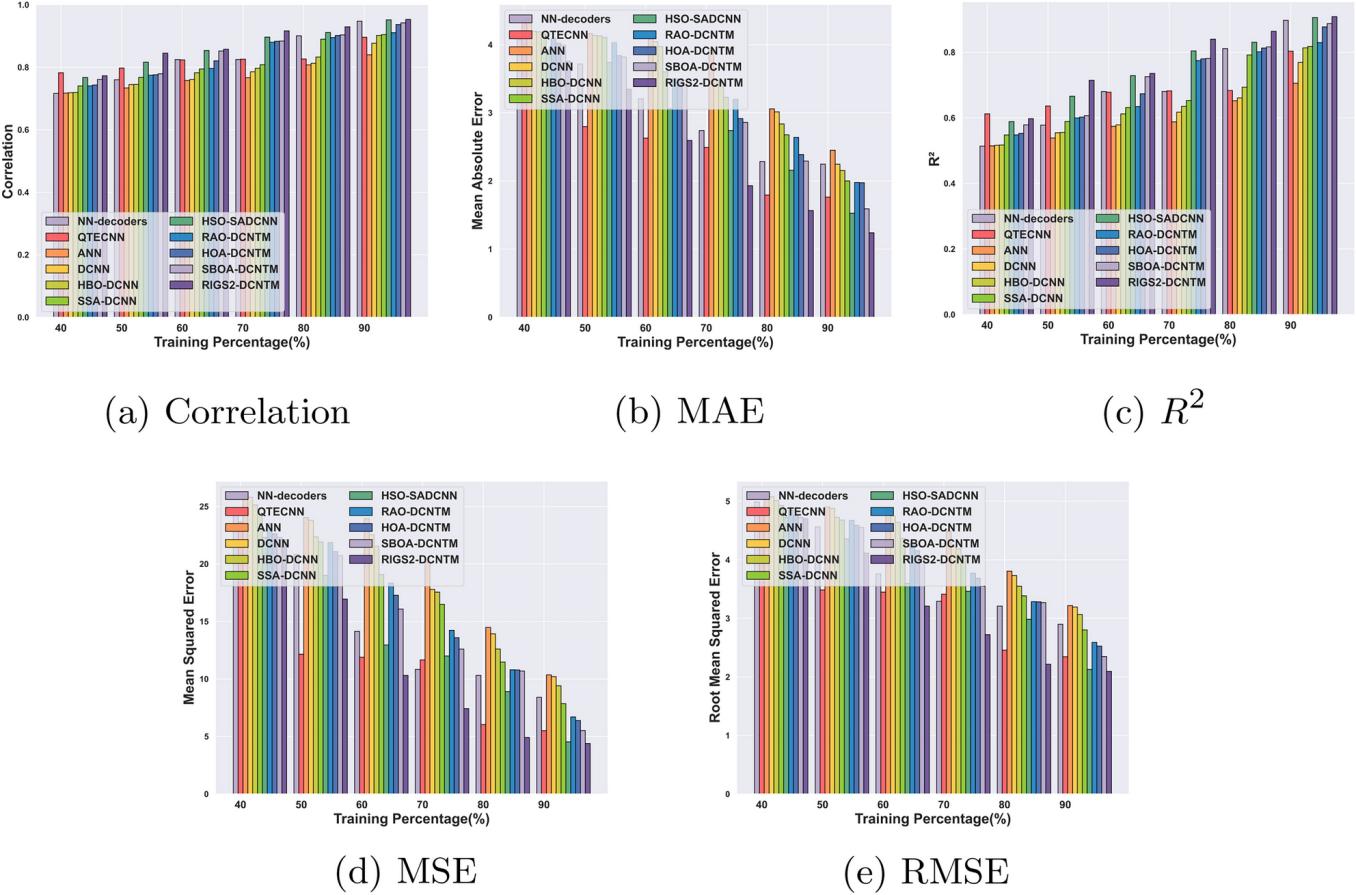
Comparative analysis for Circuit 2: (**a**) Correlation, (**b**) MAE, (**c**) $R^2$, (**d**) MSE, (**e**) RMSE.

Figure 10 shows the findings of the comparative analysis of the RlGS2-DCNTM model for circuit 2. The RlGS2-DCNTM model achieves a maximum correlation of 0.95 for 90% of training, which is shown in Fig. 10a, outperforming the current methods such as QTECNN, ANN, and DCNN with significantly better differences of 0.057, 0.11, and 0.076, respectively. As shown in Fig. 10b, the RlGS2-DCNTM model attains a minimal MAE of 1.23 with a substantial performance difference of 1.00, 0.52, 0.73, 0.35, and 1.00 compared to the conventional techniques such as NN-decoders, QTECNN, HOA-DCNTM, SBOA-DCNTM, and DCNN. With an MSE of 4.38 for 90% of training, the RlGS2-DCNTM model achieves a lower error value than the traditional SSA-DCNN by 3.46, HSO-SADCNN by 0.14, and ANN by 5.96 as depicted in Fig. 10d. Furthermore, the RlGS2-DCNTM model’s $R^2$ value is 0.91, indicating a better performance deviation of 0.01, 0.11, 0.2, and 0.14 in comparison to more conventional techniques such as NN-decoders, QTECNN, ANN, and DCNN, respectively that is mentioned in Fig. 10c. The RlGS2-DCNTM model has a lower RMSE of 2.09, indicating a notable performance divergence of 0.80 compared to NN-decoders and 0.97 compared to HBO-DCNN, which is delineated in Fig. 10e. The results demonstrate the efficacy of the proposed model in QEC, which is achieved through the utilization of statistical features, a self-spare attention mechanism, and an ensemble deep-learning model.

### Complexity analysis

**Fig. 11:**
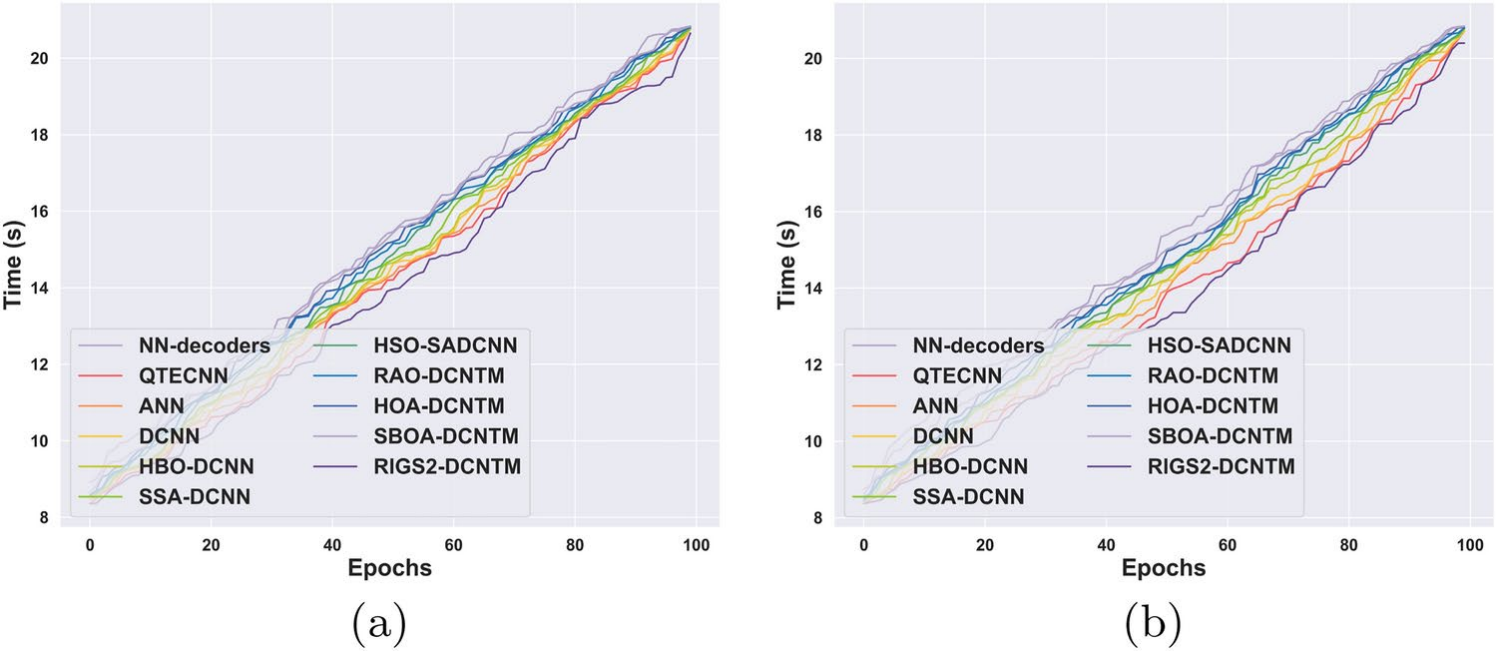
Time complexity analysis. (**a**) Computational Complexity for Circuit 1. (**b**) Computational Complexity for Circuit 2.

The time complexity analysis of the RlGS2-DCNTM decoder with the existing methods for circuits 1 and 2 is illustrated in Fig. 11a and b. The figures exhibit that the increase in epoch value is directly proportional to the time. For 20th epoch with the RlGS2-DCNTM model requires 10.09s for performing error correction, however, for the 100th epoch the time requirement is increased to 20.65 s. When compared with the existing algorithms, the RlGS2-DCNTM model takes less time for error correction. Similarly, for the second circuit, the RlGS2-DCNTM model takes less time 17.23 s for the 80th epoch, while the conventional HSO-SDCNN model takes 18.23s.

### Comparative discussion

The comparative discussion of the RlGS2-DCNTM decoder model over the existing methods is described in Tables 2 and 3. While the conventional error correction codes and decoders offer superior results, they came up with several challenges. The traditional NN-decoder models require high-quality training data, which limits the efficiency of the model. Moreover, the complexity of the hexagonal codes increased concerning the distance between the codes, which limited the real-world pertinence of the DCNN model. The QTECNN model faces issues with increased computational complexity. The HBO-DCNN model produces local optima problems. The HSO-SADCNN model did not correct dynamic errors, which impacted the resilience of the deep learning model in real time application, and the SSA-DCNN methods also produce local optima problems. Therefore, to tackle these challenges, this research designs an RlGS2-DCNTM model, which resolves the model complexity issues through a self-sparse attention mechanism. The premature convergence, slow learning rate, and local optima problems are rectified using the proposed RlGS2 model. The proposed model also mitigates the vanishing gradient problems and data requirement challenges and solves complex error codes effectively. The proposed RlGS2-DCNTM model offers superior error decoding performance with a minimum MSE of 4.26, RMSE of 2.06, and MAE of 1.14 as well as maximum correlation and $R^ 2$ of 0.96 and 0.92 respectively.

**Table 2:** Comparative Discussion of RlGS2-DCNTM model for Circuit 1.

Methods/Metrics	Correlation (%)	MSE	$R^2(\%)$	MAE	RMSE
NN-decoder	95	5.41	90	1.68	2.33
QTECNN	93	5.41	86	1.57	2.33
Data ANN	90	11.23	80	2.74	3.35
DCNN	91	8.91	83	2.35	2.99
HBO-DCNN	92	8.72	85	2.21	2.95
SSA-DCNN	93	6.34	86	1.86	2.52
HSO-SADCNN	95	6.34	90	1.39	2.12
RAO-DCNTM	93	4.98	89	1.45	2.52
HAO-DCNTM	94	4.98	89	1.45	2.23
SBOA-DCNTM	95	4.78	90	1.39	2.19
RLGS2-DCNTM	96	4.26	92	1.14	2.06

**Table 3:** Comparative Discussion of RlGS2-DCNTM model for Circuit 2.

Methods/Metrics	Correlation (%)	MSE	$R^2(\%)$	MAE	RMSE
NN-decoder	94.70	8.41	90	2.25	2.90
QTECNN	89.60	5.50	80	1.76	2.35
ANN	84	10.35	71	2.45	3.22
DCNN	87.70	10.20	77	2.25	3.19
HBO-DCNN	90.20	9.40	81	2.15	3.07
SSA-DCNN	90.40	7.86	82	2.00	2.80
HSO-SADCNN	95.20	4.53	91	1.53	2.13
RAO-DCNTM	91.10	6.70	83	1.98	2.59
HAO-DCNTM	93.70	6.38	88	1.97	2.53
SBOA-DCNTM	94.20	5.52	89	1.59	2.35
RLGS2-DCNTM	95.30	4.39	91	1.24	2.10

## Conclusion

In conclusion, this research proposes an RlGS2-DCNTM decoder model for QEC, which diagnoses errors accurately. The RlGS2-DCNTM model’s robustness in handling complex error patterns and the efficiency of the self-sparse attention mechanism for focusing relIn conclusion, this research proposes anRlGS2-DCNTM decoder model for QEC, which diagnoses errors accurately. The RlGS2-DCNTM model’s robustness in handling complex error patterns and the efficiency of the self-sparse attention mechanism for focusing relevant data points progress the decoding performance of the model. In addition, the effective feature learning and long-term dependency learning ability of the proposed model lessen the vanishing gradient problems and enhance the fault tolerance performance of the model. The RlGS algorithm tunes the hyperparameters of the proposed decoder, which improves the learning rates and convergence speed. In addition, when compared with other traditional mechanisms the proposed model offers superior error decoding performance with a minimum MSE of 4.26, RMSE of 2.06, and MAE of 1.14 as well as maximum correlation and $R^2$ of 0.96 and 0.92 respectively. Further, the proposed RlGS2-DCNTM model offers reliable and efficient decoding performance for complex quantum codes, thus paving the way for their practical applications beyond the reach of classical computing systems. In future, the proposed model can be extended to design reinforcement learning-based hybrid decoders for scalable error correction.

## Data Availability

Data are available on request to the corresponding author Ravikumar Bandaru at ravimaths83@gmail.com.
